# Effective differentiation of mild cognitive impairment by functional brain graph analysis and computerized testing

**DOI:** 10.1371/journal.pone.0230099

**Published:** 2020-03-16

**Authors:** Rok Požar, Bruno Giordani, Voyko Kavcic

**Affiliations:** 1 University of Primorska, Faculty of Mathematics, Natural Sciences and Information Technologies, Koper, Slovenia; 2 University of Primorska, Andrej Marušič Institute, Koper, Slovenia; 3 Departments of Psychiatry, Neurology, and Psychology and School of Nursing, University of Michigan, Ann Arbor, MI, United States of America; 4 Institute of Gerontology, Wayne State University, Detroit, MI, United States of America; 5 Biomedical Research Institute, Ljubljana, Slovenia; Universidad Rey Juan Carlos, SPAIN

## Abstract

Community-dwelling African American elders are twice as likely to develop mild cognitive impairment (MCI) or Alzheimer’s disease and related dementias than older white Americans and therefore represent a significant at-risk group in need of early monitoring. More extensive imaging or cerebrospinal fluid studies represent significant barriers due to cost and burden. We combined functional connectivity and graph theoretical measures, derived from resting-state electroencephalography (EEG) recordings, with computerized cognitive testing to identify differences between persons with MCI and healthy controls based on a sample of community-dwelling African American elders. We found a significant decrease in functional connectivity and a less integrated graph topology in persons with MCI. A combination of functional connectivity, topological and cognition measurements is powerful for prediction of MCI and combined measures are clearly more effective for prediction than using a single approach. Specifically, by combining cognition features with functional connectivity and topological features the prediction improved compared with the classification using features from single cognitive or EEG domains, with an accuracy of 86.5%, compared with the accuracy of 77.5% of the best single approach. Community-dwelling African American elders find EEG and computerized testing acceptable and results are promising in terms of differentiating between healthy controls and persons with MCI living in the community.

## Introduction

With the aging of the population, age- and disease-related cognitive declines have important socioeconomic implications. Identifying those who are at risk for accelerated cognitive decline and understanding the mechanisms leading to this decline are vital for guiding interventions and improving early prediction of dementia. Community-dwelling African American elders are twice as likely to develop mild cognitive impairment (MCI) or Alzheimer’s disease (AD) and related dementias compared with older white Americans and therefore represent a significant at-risk group in need of early monitoring [1]. More extensive imaging or cerebrospinal fluid studies represent significant barriers due to cost and burden. Community-based, fast, and efficient screening methods, such as portable electroencephalogram (EEG) or computer-based cognitive testing, present potential screening options to identify individuals for whom more extensive evaluations are necessary or who may be most appropriate for clinical trials aimed at early disease prevention.

Newer analysis and processing approaches for EEG provide additional options for early characterization of cognitive change. The concept of functional connectivity and graph theory, in particular, have recently been extensively applied to understand the behavior of interactions in the human brain [2–4]. In parallel to functional connectivity, which provides information on statistical dependence between activities of different regions of the brain [5, 6], graph theory offers a graph-based representation of the brain, together with a powerful framework for investigating its underlying organization. A functional EEG brain graph consists of vertices corresponding to electrodes and each pair of vertices is connected by a weighted edge, where the edge weights represent a measure of synchronization between EEG signals.

Numerous studies have reported a loss of functional connectivity in patients with AD compared to normal controls in resting state [7, 8] supporting the concept of AD as a disconnection syndrome [9]. Similar findings of a reduced level of functional connectivity have also been reported in patients with MCI [10–12], suggesting that MCI may be viewed as a disconnection syndrome, as well. In addition to functional connectivity alterations, recent studies in patients with AD show a deviation of brain graph topology from optimal small-world topology, however, the results between studies differ drastically (c.f., [13] and references therein). Perhaps unsurprisingly, changes in brain graph topology have also been described in patients with MCI [12, 14–18]. Although these results may show that brain graph topology is disrupted in MCI patients, the direction of the disruption is not clearly understood. Contradictory findings are partially due to different methodological aspects in brain graph analysis. In particular, normalization is an important step in traditional graph analysis, usually done by thresholding and/or comparing brain graph parameters to those in random graphs. However, these methods do not yield an optimal solution [19, 20]. An alternative that avoids the aforementioned limitations is to take and compare a minimum spanning tree (MST) of the brain graph—a subgraph connecting all vertices with the minimum sum of the inverse edge weights [21]. The MST represents, by definition, the backbone of the brain graph in a sense that it captures the strongest connections. Several studies have applied MST analysis to show that brain graph topology is altered in neurological diseases such as multiple sclerosis [22], Parkinson's disease [23] and Alzheimer’s disease [24]. Perhaps surprisingly, only one study used the MST approach to compare healthy and MCI groups [25]. That study found that MST topology differed between groups, however, differences did not survive correction for multiple testing. Moreover, in the same study, traditional graph measures were also used, but interestingly, these measures did not distinguish between groups. Nevertheless, additional study is necessary to understand if the MST approach is sensitive enough to detect differences between healthy and MCI groups.

An additional factor affecting the results in the above studies in terms of effectively differentiating MCI from normal could be the fact that MCI patients may show a range of cognitive impairment type and severity prior to diagnosis of dementia [26]. One option to improve the sensitivity of the comparisons could be to consider both cognitive status, as well as functional connectivity and graph-theoretic parameters derived from EEG. Various studies have employed classification approaches for the identification of MCI patients using functional connectivity and graph theory [27–30]. However, to the best of our knowledge, no study to date investigating aging and cognitive decline, and in particular differences across MCI and controls, has combined both EEG and cognitive performance measures.

Our study aimed to investigate whether resting-state EEG could detect differences in the functional connectivity and brain graph topology in MCI and whether connectivity, topological and computer-based cognitive measures can be used together in a model to distinguish which variables can best differentiate MCI patients from healthy controls. Our sample represented a group of individuals at higher risk for MCI: community-dwelling African American elders reporting a cognitive change over the past year. We hypothesized that functional connectivity brain graph topology is disrupted in MCI patients as compared to controls and that the combination of both EEG and behavioral measures improves normal/MCI classification efficiency. To test these hypotheses, functional connectivity was derived from EEG data and functional brain graphs together with their MSTs. To quantify the topological organization of these graphs, we computed the two most basic traditional graph measures that together characterize the concept of small-world topology, namely the average clustering coefficient and the average characteristic path length for weighted graphs, as well as several graph measures derived from the MST. We then compared them between the MCI and control groups. Connectivity, topological, and cognitive measures were then used to discriminate between the two groups in a model based on linear discriminant analysis.

## Material and methods

### Participants

We recruited 40 community-dwelling African American participants (36 females, four males), ranging in age from 62 to 86 years, from the greater Detroit area. Some of the participants were recruited out of the pool of over 1125 registered volunteers in the Healthier Black Elders Center, a collaboration between Wayne State University's Institute of Gerontology and University of Michigan’s Institute of Social Research [31] and others were recruited through the Michigan Alzheimer’s Disease Research Center (MADRC) from outreach programs in local churches and community centers. To evaluate a group of participants with a higher than usual expectation of an MCI diagnosis, persons were recruited if they were living in the community and considered themselves to be functioning fully, though they also responded positively to a question asking if they had experienced a decline in cognitive ability over the past year. All participants were diagnosed at the MADRC consensus conference; 27 of them being normal and 13 with MCI. All participants were consented and signed a written consent document. All procedures were in accordance with the principles expressed in the Declaration of Helsinki and approved by the Wayne State University Research Subjects Review Board and the University of Michigan Medical School Institutional Review Board.

### EEG recordings

Scalp electroencephalographic activity was recorded for at least 3 min of resting-state with eyes closed using Brain Vision (Brain Vision, Inc.) equipment. We used the high-density Acti Cap (64 active electrodes) modified according to the International 10–20 System. The recording locations included eight midline sites, with the FCz electrode as an on-line reference and a ground at midline location AFz. Low and high pass filter settings were 0.1 and 70 Hz Hz, respectively. The cutoff frequencies for these filters were set at 3 dB down; the roll-off was 12 dB per octave at both sides. Impedances were maintained below 10 kΩ for each channel and balanced across all channels within a 5 kΩ range. The sampling rate was 500 Hz with a 32-bit resolution.

### EEG data analyses

Resting-state eyes-closed EEG was off-line inspected to identify, and segments of EEG contaminating either excessive noise, saturation, or lack of EEG activity were removed. The EEG data were then segmented in consecutive epochs of 2 seconds and were analyzed off-line (1024 data points; 0.488 Hz resolution; Hanning window). The epochs were identified as acceptable by an automatic computerized procedure, using a rejection criterion of 100 mV on any channel affected by artifacts (muscular, instrumental). Per subject, we obtained on average 90 (range, 64–115) 2 seconds of artifact-free segments.

A total of twelve regions of interest (ROI) were selected for further analysis: Right frontal—RF (Fp1, AF7, AF3, F7, F5, F3), Medium frontal—MF (F1, Fz, F2, FC1, FC2), Left frontal—LF (F4, Fp2, AF4, AF8, F6, F8), Left temporal—LT (FT9, FT7, T7, TP7, TP9), Left central—LC (FC5, FC3, C5, C3, CP5, CP3), Medial central—MC (C1, Cz, C2, CP1, CPz, CP2), Right central—RC (FC4, FC6, C4, C6, CP4, CP6), Right temporal—RT (FT10, FT8, T8, TP8, TP10), Left parietal—LP (P7, P5, P3, PO7, PO3), Medial parietal—MP (P1, Pz, P2, POz), Right parietal—RP (P4, P6, P8, PO4, PO8), occipital—O (PO9, O1, Oz, O2, PO10); see Fig 2.

### Computerized neuropsychological assessment

Two standardized, laptop computerized neuropsychological screening batteries, the Brief CogState Battery and the NIH Toolbox-Cognition were chosen to assess specific aspects of cognitive functioning. It was expected that the two batteries, together, might provide the most sensitive coverage of basic domains of cognition. Also, these measures are easily administered and highly portable and therefore suitable for use in community settings.

#### CogState battery

CogState has been demonstrated to be reliable, stable and resistive to practice effects [32, 33] and sensitive to both MCI [34, 35] and early cognitive changes in healthy controls [32, 36]. The following subtests comprising the Brief CogState Battery were administered: Detection Task (DET, speed of processing), Identification (IDN, attention), One Back-Working Memory (ONB, working memory), and One Card Learning (OCL; learning/memory). The test battery requires approximately 12–15 minutes to complete. Specific log-transformed measures are available for each task based on response times and accuracy (c.f., [35, 37] for more detail).

#### NIH Toolbox-Cognition

The NIH Toolbox-Cognition was developed through the NIH Blueprint for Neuroscience Research as a computer-based assessment program with an emphasis on measuring outcomes in longitudinal epidemiologic studies and prevention or intervention trials. The battery has been normed and validated across the lifespan in subjects ages 3–85. The following subtests of the NIH Toolbox-Cognition were administered: Picture Vocabulary Test (PVT), Oral Reading Recognition Test (ORRT), List Sorting Working Memory Test (LSWMT), Dimensional Change Card Sorting Test (DCCST), Pattern Comparison Processing Speed Test (PCPST), and Picture Sequence Memory Test (PSMT). The first two measures make up the more static Crystalized subtests reflecting premorbid ability and the remaining the more sensitive Fluid measures. Scores are fully adjusted for demographics of age, education, race and presented as T-Scores with a mean of 50 and a standard deviation of 10. A recent report [38] describes the development of the Toolbox in addition to results on test-retest reliability, age effects on performance, and convergent and discriminant construct validity. The completed test battery takes approximately 30–35 minutes and was administered on a laptop computer.

### Functional connectivity analysis

The *phase lag index* (PLI) was used as a measure of functional connectivity [39]. It measures the asymmetry of the distribution of instantaneous phase differences between two time series *x*_*i*_(*t*) and *x*_*j*_(*t*) (*t* = 1,2,…,m):

$$\text{PLI}(x_{i},x_{j})=\left|1/m\sum_{t=1}^{m}\text{sign}\left(\varphi_{i}\left(t\right)–\varphi_{j}\left(t\right)\right)\right|,$$

where sign(x) is the sign function, and *φ*_*i*_(*t*), *φ*_*j*_(*t*) are the instantaneous phases associated with *x*_*i*_(*t*) and *x*_*j*_(*t*), respectively. For further analysis, the PLI was computed between all pairs of 64 electrodes for each epoch in the following frequency bands: delta (0.5–4 Hz), theta (4–8 Hz), lower alpha (8–10 Hz), upper alpha (10–13 Hz) and beta (13–30 Hz). The result of computations was a 64 × 64 PLI matrix in which (*i*,*j*)-cell stores the value PLI(*x*_*i*_,*x*_*j*_). For every subject, the average PLI matrix was calculated over all epoch.

The analysis was done at the global and local levels. To measure the global functional connectivity *the global average* PLI was computed as the average over off-diagonal elements of the average PLI matrix for each frequency band. Only if significant group differences were observed in the global average PLI for a specific frequency band, a local analysis was performed at the level of the ROI. Based on twelve ROI, *the regional average* PLI of each region *R* was computed as the average over those off-diagonal elements of the average PLI matrix that belong to pairs of electrodes lying in *R*. Besides, *the regional* PLI between each pair of regions *R* and *L* was calculated as the average over those off-diagonal elements of the average PLI matrix that belong to pairs of electrodes of which one lies in *R* and another in *L*. The construction of PLI matrices was performed using *BrainWave* software (version 0.9.152.41, available from http://home.kpn.nl/stam7883/brainwave.html). Global and local functional connectivity analysis, as well as subsequent analysis of graph topology, were performed in *Matlab v2011*.

### Graph theory analysis

In our study, a *brain graph*, uniquely represented by the PLI matrix, consists of vertices representing electrodes, and each pair of vertices is connected by a weighted edge with the weight equal to the PLI between the corresponding electrodes. Brain graphs associated with the average PLI matrices were used for further analysis. To characterize brain graph topology, we applied a traditional graph analysis and MST approach.

#### Traditional graph analysis

From the average PLI matrices, the average weighted clustering coefficient and the average weighted characteristic path length were calculated. The weighted clustering coefficient of a node *v*_*i*_ in a weighted graph with the set of vertices *V* = {*v*_1_,*v*_2_,…,*v*_n_} is defined as

$$C_{v_{i}}^{w}=\frac{\sum_{\begin{array}{c} v_{k},v_{i}\in V\\ v_{k}\neq v_{i} \end{array}}\sum_{\begin{array}{c} v_{i},v_{k},v_{l}\in V\\ v_{l}\neq v_{i},v_{l}\neq v_{k} \end{array}}w_{i,k}w_{i,l}w_{k,l}}{\sum_{\begin{array}{c} v_{k},v_{i}\in V\\ v_{k}\neq v_{i} \end{array}}\sum_{\begin{array}{c} v_{i},v_{k},v_{l}\in V\\ v_{l}\neq v_{i},v_{l}\neq v_{k} \end{array}}w_{i,k}w_{i,l}},$$

where *w*_*i*_,_*j*_ denotes the weight of the edge from *v*_*i*_ to *v*_*j*_. The average weighted clustering coefficient is then computed as

$$C^{w}=1/n\sum_{i=1}^{n}C_{v_{i}}^{w}.$$

The length of a path in a weighted graph is the sum of weights of the edges in the path. As for brain graphs, the weights were defined as the inverses of the average PLI values. Then a shortest path between two vertices *u* and *v* is defined as that path which has the shortest length among all the paths connecting *u* and *v*. The length of a shortest path from *u* to *v* is denoted by *d*(*u*,*v*). The average weighted characteristic path length is computed as

Lw=1n(n−1)∑u∈V∑v∈Vu≠vd(u,v).

To reduce the effect of edge weights, the average weighted clustering coefficient and the average weighted characteristic path length were normalized by random brain graphs. To this end, fifty random brain graphs were created by randomly reshuffling the original values in each average PLI matrix, and the values $\langle C^{w}\rangle$ and $\langle L^{w}\rangle$ were computed as the average weighted clustering coefficient and the average weighted characteristic path length, respectively, averaged over all fifty random graphs. Finally, the normalized average weighted clustering coefficient C^w=Cw〈Cw〉 and the normalized average weighted characteristic path length L^w=Lw〈Lw〉 were used in further analyses.

To identify if brain graphs show a small-world property, the small-world index was calculated as. G with *S* > 1 are considered as graphs with a small-world topology.

#### Minimum spanning tree analysis

An MST of a brain graph is a subgraph that connects all vertices such that the sum of all its edge weights is maximized. We used Kruskal’s algorithm [40] to compute MSTs. For further characterization, the edge weights of the obtained trees were ignored.

First, the dissimilarity between MSTs of patients and controls was computed for each frequency band. Following [41], the *dissimilarity D*_(*G*|*H*)_ between two trees *G* and *H* with the same set of vertices {*v*_1_,*v*_2_,…,*v*_n_} measures how much information is needed to transform *G* to *H*:

D(G|H)=1n∑i=1nlog10|∑vj∈NH(vi)dG(vi,vj)|NH(vi)||,

where *N*_H_(*v*) denotes the set of neighbors of *v* and *d*_*G*_(*u*,*v*) is the length of a shortest path from *u* to *v* in *G*. To compute dissimilarity for both groups the MST obtained from the average PLI matrix of all controls was used as a reference. Second, only if we found significant group differences in the dissimilarity for a specific band, we performed posthoc analyses on the following topological properties of the MST: the maximum normalized vertex degree, the average normalized vertex eccentricity, the maximum normalized vertex betweenness centrality, degree divergence, normalized diameter, normalized leaf fraction, and tree hierarchy. An overview of measure definitions is given in Table 1.

**Table 1:** Measures on a tree with the set of vertices *V* = {v_1_,v_2_,…,v_n_}.

Symbol	Definition
k*(*v*)	*Normalized vertex degree*. The number of neighbors of *v*, normalized by the maximal possible number of neighbors: k*(v)=1nk(v).
k*_max_	*Maximum normalized vertex degree*. The greatest normalized vertex degree of all vertices: $k^{*}{}_{\text{max}}={\text{max}}_{v\in V}k^{*}\left(v\right)$.
*e*(*v*)	*Vertex eccentricity*. The distance of a vertex *v* to a vertex farthest from *v*: $e\left(v\right)={\text{max}}_{u\in V}d\left(u,v\right)$.
e*(*v*)	*Normalized vertex eccentricity*. The eccentricity of *v*, normalized by the maximal possible distance: e*(v)=1n−1e(v).
e*_avr_	*Average normalized vertex eccentricity*. The average normalized vertex eccentricity of all vertices: e*avr=1n∑v∈Ve*(v).
BC(*v*)	*Vertex betweenness centrality*. Measures the extent to which a given vertex is situated in paths between pairs of vertices: $\text{BC}\left(v\right)=\sum_{\begin{array}{c} u,w\in V\\ u\neq w\neq v \end{array}}\frac{\sigma (u,w|v)}{\sigma \left(u,w\right)},$ where *σ*(*u*, *w*|*v*) is the number of all shortest paths between *u* and *w* passing *v*, and *σ*(*u*, *w*) is the number of all shortest paths between *u* and *w*.
BC*(*v*)	*Normalized vertex betweenness centrality*. The betweenness centrality of *v*, normalized by the maximal possible betweenness centrality: BC*(v)=2(n−1)(n−2)BC(v).
${\text{BC}}^{*}{}_{\text{max}}$	*Maximum normalized betweenness centrality*. The greatest normalized vertex betweenness centrality of all vertices: ${\text{BC}}^{*}{}_{\text{max}}={\text{max}}_{v\in V}\text{BC}\left(v\right)$.
l*	*Normalized leaf fraction*. The number of leaves, normalized by the maximal possible number of leaves: l*=ln−1.
κ	*Degree divergence*. Measures the broadness of the degree distribution: $\kappa =\frac{\sum_{v\in V}k{\left(v\right)}^{2}}{\sum_{v\in V}k{\left(v\right)}^{2}}$.
*D*	*Diameter*. The greatest distance between any two vertices: $\text{D}={\text{max}}_{u,v\in V}\text{d}\left(u,v\right)$.
D*	*Normalized diameter*. The diameter, normalized by the maximal possible diameter: D*=1n−1D.
*T*_*h*_	*Tree hierarchy*. Measures the balance between diameter reduction and overload prevention: Th=l*2BC*max.

Two extreme situations are of particular interest in the topological characterization of a tree on *n* vertices; a *star graph* consisting of a central vertex of degree *n* − 1 and *n* − 1 leaves on the one hand, and a *path graph* consisting of *n* − 2 vertices of degree 2 and two leaves on the other hand. A star graph has centralized topology and the smallest possible diameter of 2, thus sharing the basic characteristics of random graph topology. In contrast, a path graph has decentralized topology and the largest possible diameter of *n* − 2, thus sharing the basic characteristics of regular graph topology.

### Statistical analysis

To assess differences in functional connectivity and brain graph topology between groups non-parametric randomization/permutation testing (in this study 10000 permutations) was used, since the data were generally not normally distributed [42]. All the statistical analyses were performed in *Matlab v2011*.

#### Functional connectivity

For each frequency band the global average PLI results between groups were compared with a permutation test. The value used for significance was set to P < 0.05. If there was a significant difference in the global average PLI in a specific frequency band, we performed posthoc analysis at the local level. The regional average PLI and the regional PLI were tested between groups using permutation tests and the significance level for the obtained *P* values was set to 0.01.

#### Brain graph topology

For each frequency band between-group differences in ${\hat{C}}^{w},{\hat{L}}^{w}$, and *S* were tested using a permutation test. The value used for significance was set to P < 0.05 and a correction for multiple comparisons over the three measures was performed by the false discovery rate [43].

The comparison of MST trees was carried out at two different levels. First, for each frequency band tree dissimilarity measures between groups were compared with a permutation test. The value used for significance was set to P < 0.05. Then, if there was a significant difference in dissimilarity in a specific frequency band, we performed posthoc analysis on topological properties of MSTs. Between-group differences in k*_max_, e*_avr,_ BC*_max_, l*(*v*), *κ*, D* and *T*_*h*_ were tested using permutation tests. The value used for significance was set to P < 0.05, and a correction for multiple comparisons over the seven MST measures was performed by the false discovery rate.

### Classification

**Fig 1:**
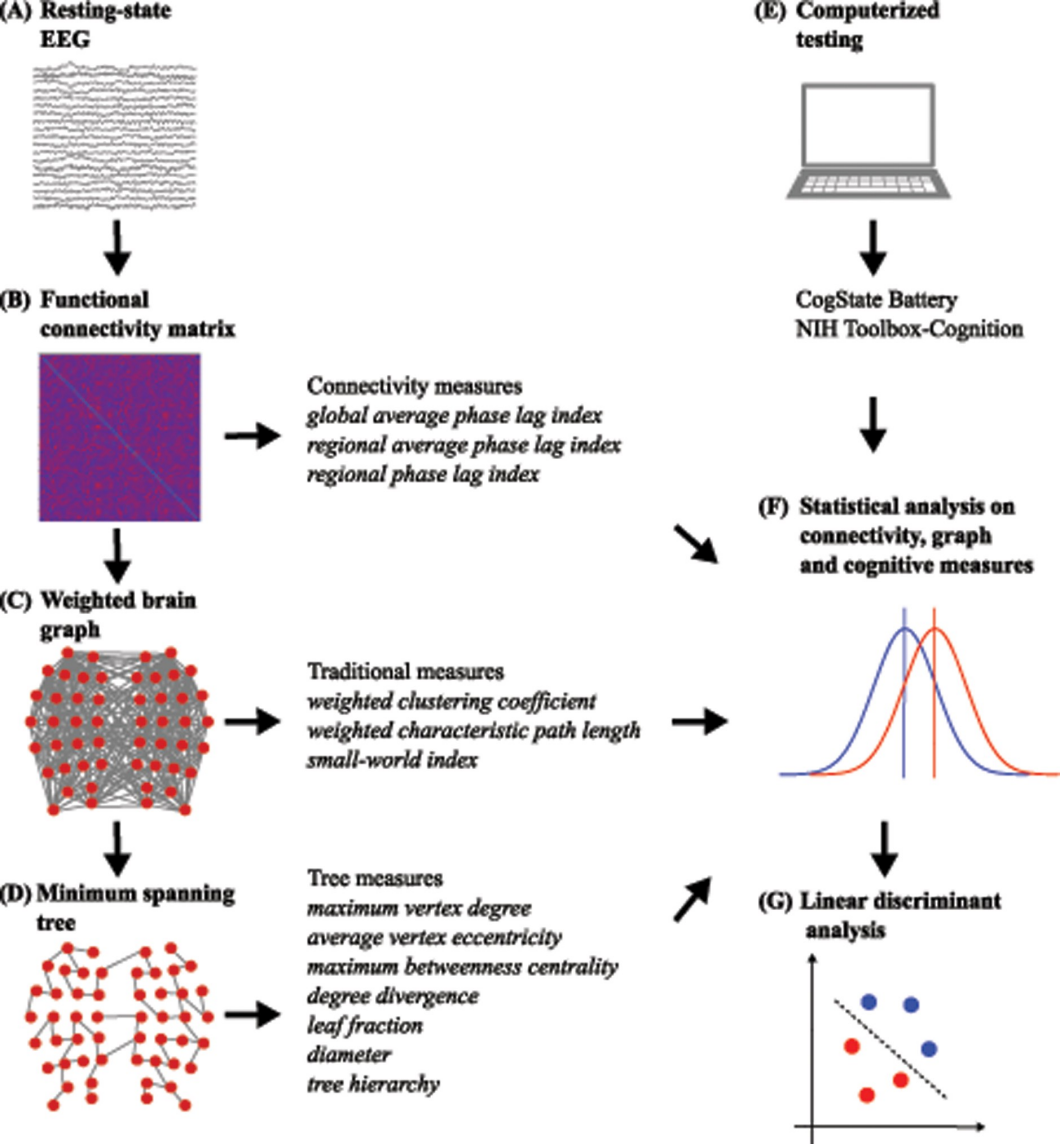
Schematic illustration of the proposed method in this study. (A) For each subject, resting-state EEG data were acquired. (B) A functional connectivity matrix was obtained from the EEG time-series data by computing the phase lag index. From the connectivity matrix, global and local connectivity measures were derived. (C) The connectivity matrix uniquely represents a brain graph, from which traditional measures were computed. (D) A minimum spanning tree of the brain graph was constructed and several tree measures were computed. (E) Two laptop computerized neuropsychological screening batteries, CogState and NIH Toolbox-Cognition were chosen to assess specific aspects of cognitive functioning. (F) Statistical differences between controls and MCIs in functional connectivity, traditional measures, MST measures, and cognitive tests were computed. (G) The obtained significant between-group differences were used as features in a stepwise linear discriminant analysis.

A linear discriminant analysis [44] was used to classify patients versus controls. The following were used as features for the analysis: (i) significant between-group differences of local functional connectivity (extracted from those frequency bands where there were significant differences between groups in the global average PLI), (ii) significant between-group differences of brain graph topology and (iii) significant between-group differences of cognitive subtest scores. First, the set of cognitive tests and the set of local functional connectivity, together with topology measures, were used individually, and classification was performed utilizing these features. Then, we combined features from both sets to verify whether the combined classification is better than prediction obtained on the individual set. To overcome the overfitting problem due to the many predictors, we used a stepwise variable selection method in each model. A leave-one-out cross-validation method was used to estimate the performance of each model. The performance was evaluated using accuracy, sensitivity (defined as the number of correctly classified MCI patients divided by the number of all MCI patients) and specificity (defined as the number of correctly classified healthy controls patients divided by the number of all healthy controls). The classification was performed using *SPSS v25*. In Fig 1, we summarized the framework of our proposed method.

## Results

The demographic characteristics of participants and individual cognitive tests in each battery are given in Table 2.

**Table 2:** Demographic characteristics and computerized neuropsychological tests of control and mild cognitive impairment subject groups.

	Controls (*N* = 27)		MCI (*N* = 13) SD		
	Mean	SD	Mean	SD	*P* value*
Age (years)	73.0741	6.9333	73.6154	5.9096	0.8848
Years of education	15.1111	2.2927	14.2308	2.2418	0.2563
Gender (% female)	93%	-	85%	-	-
CogState test					
DET	402.09	92.22	444.39	166.939	0.6614
IDN	577.57	99.99	693.81	178.90	**0.0241**
ONB	0.94	0.06	0.85	0.09	**0.0031**
OCL	0.9828	0.08	0.59	0.09	**0.0067**
Toolbox tests					
PVT	58.16	8.08	53.10	10.63	0.1047
ORRT	56.05	6.29	52.23	6.13	0.0855
LSWMT	51.73	9.82	45.36	6.20	**0.0336**
DCCST	56.15	9.94	48.76	12.30	**0.0231**
PSMT	48.33	12.43	40.13	5.51	**0.0426**
PCPST	50.55	12.87	43.23	9.75	0.1155

DET, Detection Task; IDN Identification; ONB, One Back-Working Memory; OCL, One Card Learning; PVT, Picture Vocabulary Test; ORRT, Oral Reading Recognition Test; SWMT, List Sorting Working Memory Test; DCCST, Dimensional Change Card Sorting Test; PSMT, Picture Sequence Memory Test; PCPST, Pattern Comparison Processing Speed Test; SD, standard deviation; MCI, mild cognitive impairment.

*The *P* value was obtained using Mann-Whitney U-tests for independent samples.

Bold = significant difference between the two groups.

### Functional connectivity

**Global analysis.** The global average PLI was significantly lower in MCI patients in the delta band; see Table 3.

**Table 3:** Group descriptive for the global average PLI measure.

Frequency band	Controls (*N* = 27)		MCI (*N* = 13)		
	Mean	SD	Mean	SD	*P* value
delta	0.1399	0.0081	0.1344	0.0098	**0.0439**
theta	0.1329	0.0250	0.1267	0.0189	0.4038
lower alpha	0.2433	0.0963	0.2048	0.0609	0.1496
upper alpha	0.1604	0.0371	0.1713	0.0596	0.5609
beta	0.0738	0.0091	0.0725	0.0087	0.6770

SD, standard deviation; MCI, mild cognitive impairment.

Bold = significant difference between the two groups.

#### Local analysis

**Fig 2:**
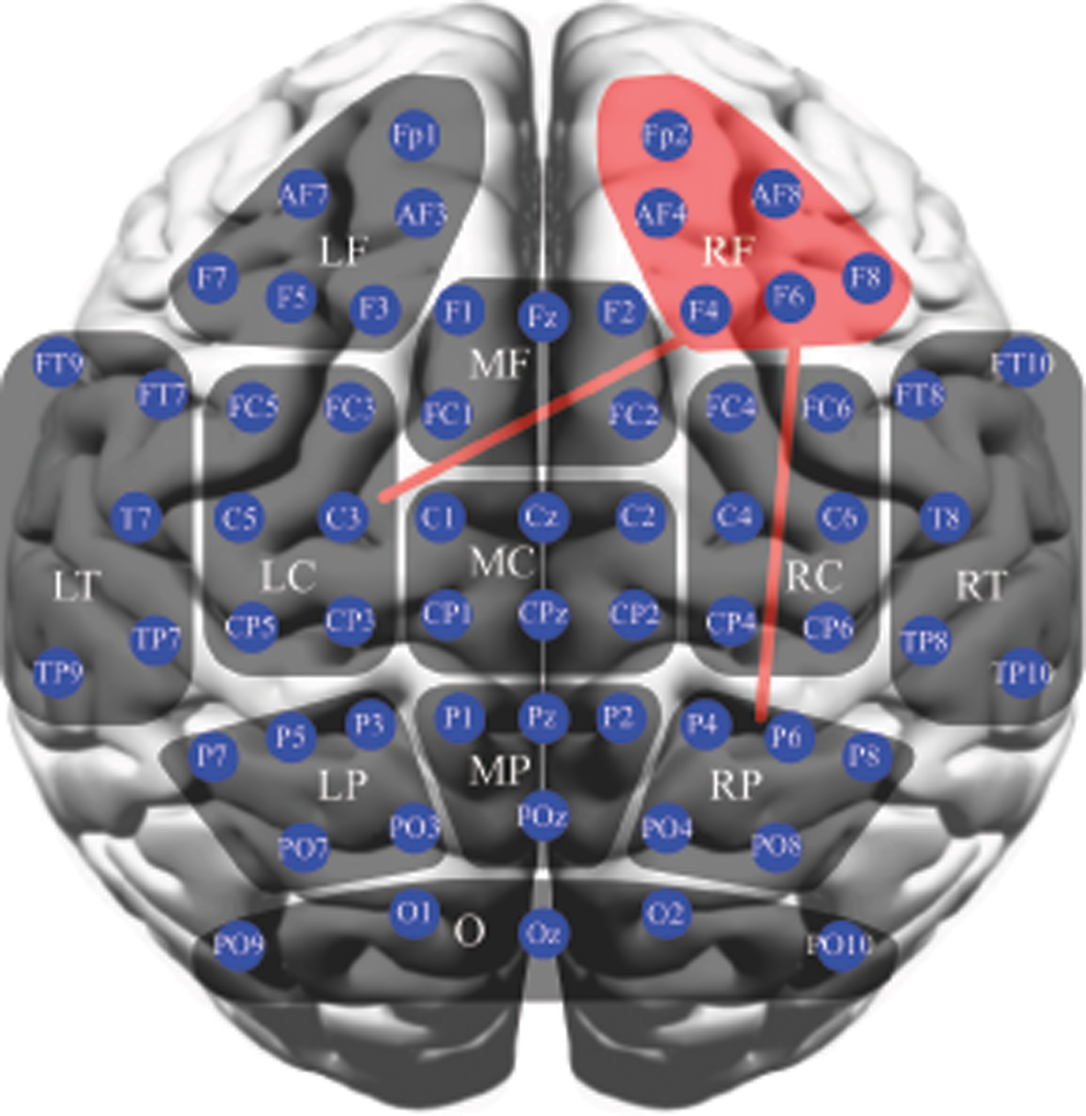
Schematic illustration of statistical analyses for regional average PLI (non-parametric permutation test; the significant difference is indicated by red region) and regional PLI (non-parametric permutation test; the significant difference is indicated by red line) in the delta band. The regional average PLI significantly decreased over the right frontal region in MCI patients. Also, the regional PLI significantly decreased between the right frontal and left central, and between right frontal and right parietal regions.

In subsequent connectivity analysis, the regional average PLI values of 12 regions were overall lower in patients in the delta band, with significance for the RF region (*P* = .0014). Also, the regional PLI values between pairs of the 12 regions of interest were overall lower in MCI participants, reaching significance for pairs between RF and LC regions and RF and RP regions (*P* = 0.0036 and *P* = 0.0020, respectively); see Fig 2.

### Graph theory analysis

#### Traditional measures

No significant differences between patients and controls were found for ${\hat{C}}^{w},{\hat{L}}^{w}$, and *S*, see Table 4.

**Table 4:** Group descriptive for traditional measures.

	Controls (*N* = 27)		MCI (*N* = 13)		
	Mean	SD	Mean	SD	*P* value
delta					
${\hat{C}}^{w}$	1.0061	0.0041	1.0043	0.0023	0.0751
${\hat{L}}^{w}$	1.0004	0.0013	1.0001	0.0004	0.2166
*S*	1.0056	0.0031	1.0042	0.0020	0.0770
theta					
${\hat{C}}^{w}$	1.0090	0.0087	1.0060	0.0047	0.1957
${\hat{L}}^{w}$	1.0015	0.0040	1.0005	0.0007	0.3387
*S*	1.0075	0.0063	1.005	0.0040	0.2462
lower alpha					
${\hat{C}}^{w}$	1.0198	0.0164	1.0152	0.0145	0.3814
${\hat{L}}^{w}$	1.0128	0.0248	1.0029	0.0111	0.0903
*S*	1.0071	0.0145	1.0125	0.0163	0.3479
upper alpha					
${\hat{C}}^{w}$	1.0121	0.0148	1.0136	0.0171	0.8035
${\hat{L}}^{w}$	1.0071	0.0219	1.0096	0.0250	0.6036
*S*	1.0052	0.0097	1.0042	0.0088	0.7714
beta					
${\hat{C}}^{w}$	1.0121	0.0151	1.0080	0.0054	0.2514
${\hat{L}}^{w}$	1.0053	0.0127	1.0010	0.0018	0.1491
*S*	1.0068	0.0035	1.0070	0.0055	0.8989

${\hat{C}}^{w}$, normalized average weighted clustering coefficient; ${\hat{L}}^{w}$, normalized average weighted characteristic path length; *S*, small-world index; MCI, mild cognitive impairment.

#### Tree dissimilarity

A significant MST dissimilarity between patients and controls was found in the delta band (*P* = 0.0015).

#### Minimum spanning tree measures

For the delta band, $k^{*}{}_{\text{max}}$, l* and κw were significantly lower in patients. Group effects on $e^{*}{}_{\text{avr}}$ and D* were significantly higher in patients. Results and statistics are summarized in Table 5.

**Table 5:** Minimum spanning tree descriptives in the delta band.

Measure	Controls (*N* = 27)		MCI (*N* = 13)		
	Mean	SD	Mean	SD	*P* value
$k^{*}{}_{\text{max}}$	0.1769	0.0715	0.1232	0.0284	**0.0017**
$e^{*}{}_{\text{avr}}$	0.1550	0.0372	0.1817	0.0221	**0.0102**
${\text{BC}}^{*}{}_{\text{max}}$	0.7341	0.0956	0.6905	0.0721	0.1194
*κ*	3.6837	0.9523	3.0417	0.2523	**0.0015**
D*	0.2010	0.0494	0.2344	0.0326	**0.0198**
l*	0.5720	0.0517	0.5324	0.0314	**0.0054**
*T*_*h*_	0.3937	0.0458	0.3895	0.0471	0.7874

$k^{*}{}_{\text{max}}$, maximum normalized vertex degree; $e^{*}{}_{\text{avr}}$, average normalized vertex eccentricity; ${\text{BC}}^{*}{}_{\text{max}}$, maximum normalized vertex betweenness centrality; κ, degree divergence; D*, normalized diameter; l*, normalized leaf fraction; *T*_*h*_, tree hierarchy; MCI, mild cognitive impairment.

Bold = significant after correcting for the seven global minimum spanning tree measures by false discovery rate.

### Classification

**Fig 3:**
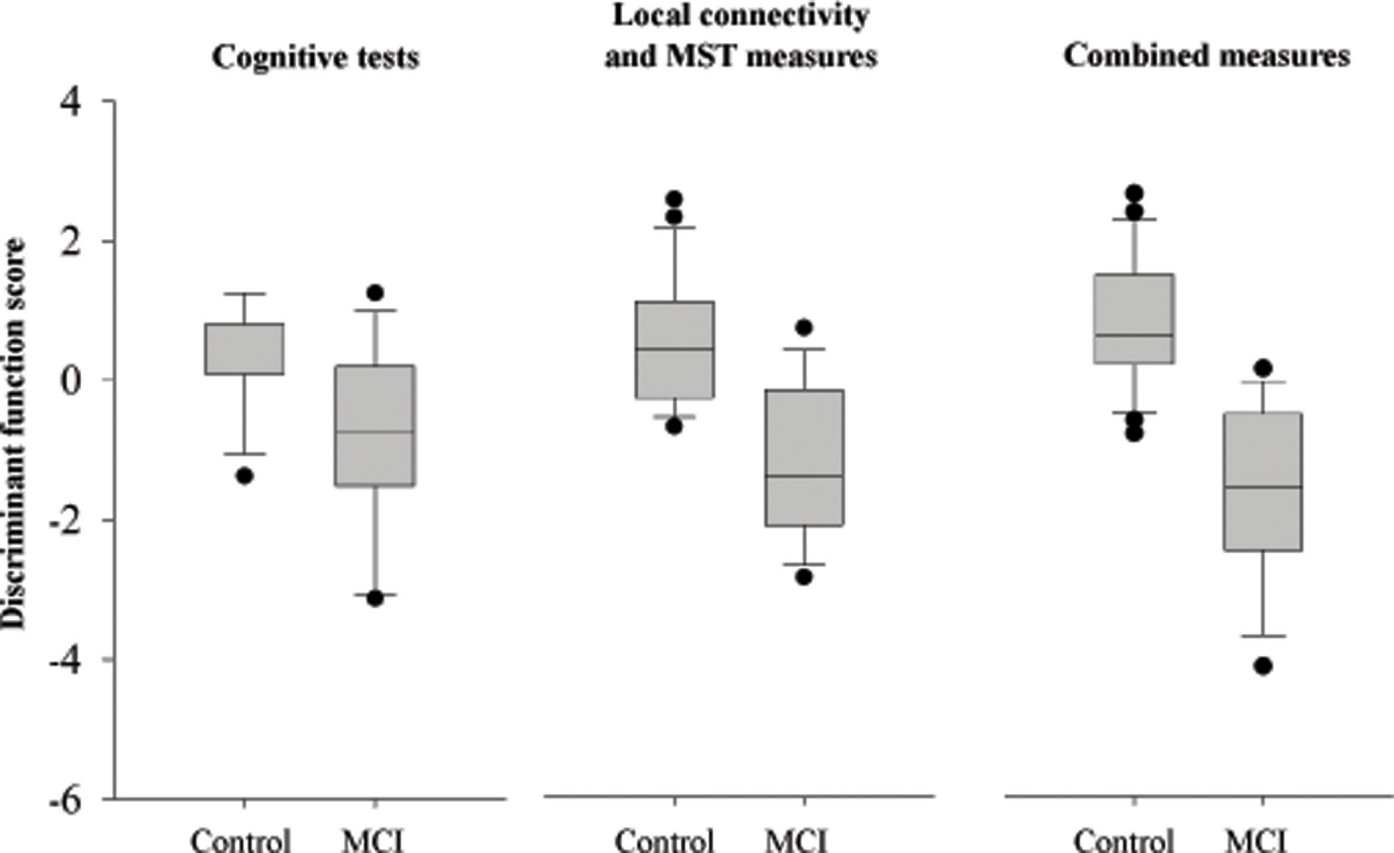
Box plots of each classification model illustrate the distribution of the discriminant function scores for the control and MCI groups.

Given the significant between-group differences of local functional connectivity, MST topology in the delta band and the significant between-group differences of cognitive tests, we tested whether these measures could be used as features to classify MCI patients versus controls using linear discriminant analysis. The outcomes for all three classification models, namely the one based on cognitive tests, the one based on functional connectivity and MST topology measures, and the combined one are shown in Table 6. The performance of each model is given in Table 7. Combined features classified our participants as controls vs MCI noticeably better than features from single cognitive or EEG domains (86.5% vs 72.2%, 77.5%, respectively). Box plots of discriminant score distributions are shown in Fig 3.

**Table 6:** Wilks’ λ and selected features of linear discriminant analyses.

Model	Wilks’ λ	*Χ*^2^	*df*	*P* value	Selected features
Cognitive tests	0.719	10.394	1	0.001	ONB
Local functional connectivity and MST topology measures	0.587	19.079	2	< .001	$k^{*}{}_{\text{max}}$
Combined measures	0.458	23.821	3	< .001	OCL
					RF-RP regional PLI
					$k^{*}{}_{\text{max}}$

ONB, One Back-Working Memory; RF-RP regional PLI, regional PLI between right frontal and right parietal regions; $k^{*}{}_{\text{max}}$, maximum normalized vertex degree, OCL, One Card Learning.

**Table 7:** Overview of classification results.

Model	Accuracy	Sensitivity	Specificity
Cognitive tests	0.722	0.417	0.875
Local functional connectivity and MST topology measures	0.775	0.538	0.889
Combined measures	0.865	0.667	0.96

## Discussion

We addressed the question of whether we could detect differences in EEG functional connectivity and brain graph organization between MCI and healthy controls by applying the PLI and the MST analysis. We were able to identify global and local differences in functional connectivity and MST topology between the two groups. Then, we showed that these abnormalities in combination with cognitive tests, if used as features in a classification model, can predict best whether a person belongs to the MCI or healthy control groups with high accuracy.

We found that the global average PLI was decreased in MCI patients in the delta band. Post-hoc analysis revealed that the regional average PLI significantly decreased over the right frontal region in patients, and the regional PLI decreased between the right frontal and left central, and between right frontal and right parietal regions. Parietal and frontal cerebral areas are particularly sensitive to brain changes very early on among participants with cognitive impairment. Our findings are in agreement with previous EEG studies. In MCI patients the decrease of synchronization likelihood in the delta band was most apparent between the right fronto-parietal regions [45]. Similar results were also reported in amnestic MCI patients using PLI [11]. The current study thus supports the hypothesis that MCI may be viewed as a disconnection syndrome, as well.

Besides the functional connectivity, we also studied brain graph topology. We started with two traditional graph measures. The simplest and most commonly used measure that reflects global integration in a graph is the characteristic path length. It is defined as the minimum number of edges required to traversed from one vertex to another, on average [19]. Another traditional measure is the clustering coefficient that describes local specialization in a graph. It measures the average probability that two vertices, having a common neighbor, are themselves connected by an edge. These two measures are sufficient to distinguish random graphs from regular graphs. In random graphs, the characteristic path length and the clustering coefficient are low, whereas regular graphs are associated with long characteristic path length and high clustering coefficient [46]. Previous studies have revealed that healthy brain graphs show the topology of small-world graphs [2, 47, 48]; the class of graphs that lies between random and regular graphs. On the one hand, small-world graphs possess a high level of local integration, such as regular graphs, while on the other hand, a high level of global efficiency, such as random graphs. We found that brain graphs of MCI and healthy controls all showed the small-world property and that the clustering coefficient and the characteristic path length did not differ between the two groups. Other studies, however, have presented controversial results in terms of the characteristic path length and the clustering coefficient. For example, these two measures did not distinguish between groups in a recent magnetoencephalography study as well [25]. Next, a recent diffusion tensor imaging study found longer characteristic path length and a higher clustering coefficient in the amnestic MCI group [18], indicating more regular topology. Similarly, a functional brain graph study reported abnormally increased characteristic path length with preserved clustering coefficient in amnestic MCI [14]. On the contrary, another magnetoencephalography study found shorter characteristic path length in MCI patients and thus a shift towards more random organization [49].

In addition to traditional graph analysis, we also used the MST approach to characterize brain graph topology. Our results revealed changes in a tree dissimilarity between patients with MCI and controls in the delta band. Subsequent post-hoc analysis showed lower vertex degree, degree divergence and leaf fraction, and higher average vertex eccentricity and diameter for MCI patients, reflecting a less integrated graph topology in this frequency band. The results showed that the MST topology of MCI patients tends to deviate from a more centralized star-like topology towards a more decentralized path-like topology and that the MST approach is more sensitive to detect the subtle differences between healthy and MCI groups than the traditional graph approach.

An interesting consideration that is relevant to the interpretation of our findings is the relationship between MST measures and traditional graph measures. To date, the empirical evidence has been somewhat limited, but studies that compared MST measures to the traditional ones provided contradictory results, indicating that topological changes of MST cannot be easily explained in terms of changes in the characteristic path length and the clustering coefficient. A study of brain graphs in children identified that a more path-like MST with longer diameter and smaller leaf fraction relates to a more regular graph topology with longer characteristic path length and higher clustering coefficient, while a more star-like MST with shorter diameter and higher leaf fraction relates to a more random graph topology with shorter characteristic path length and smaller clustering coefficient [50]. This is in agreement with a simulation study which demonstrated that the characteristic path length and the clustering coefficient have a strong positive correlation with the MST diameter, but both have a strong negative correlation with the MST leaf fraction when the underlying graph topology is changed from a random to a regular one [51]. More precisely, the diameter was short and the leaf fraction was high for MST derived from random graph topology, while the diameter increased and the leaf fraction decreased as graph topology became more regular. However, a study on graph topology in Parkinson’s disease gives evidence for the contrary, namely a more path-like MST with longer diameter and smaller leaf fraction corresponded to a more random graph topology with shorter characteristic path length and smaller clustering coefficient [23]. For this reason, we performed an additional analysis to check whether the strong correlations reported in [51] are evident in our data (S1 Table). We found that the characteristic path length and the diameter were positively correlated but not significant, while the clustering coefficient and the diameter were strongly negatively correlated which is not in agreement with Tewarie and co-workers [51]. Moreover, the characteristic path length and the leaf fraction were positively correlated but not significant, while the clustering coefficient and the leaf fraction were strongly positively correlated which is again in conflict with Tewarie and co-workers [51]. A possible reason for this is that MST does not contain cycles, and so there is probably no direct relationship to the clustering coefficient. Consequently, our significant findings in MST parameters cannot be simply compared with studies based on traditional graph measures, and therefore we cannot conclude that our results show a more regular topology in the MCI group.

By combining cognitive tests with measurements of functional connectivity and MST topology, the prediction accuracy of the model was improved by almost 10% compared to a single approach. It is not at all surprising that One Card Learning, the regional PLI between right frontal and right parietal regions and degree divergence were found to be the most important predictors for the equation. First, the regional PLI measures functional connectivity between different regions of the brain. As already mentioned, various studies have confirmed that functional connectivity is reduced in MCI and AD patients compared to controls. Next, One Card Learning is the primary learning measure included in the CogState Brief Battery. Learning measures are the primary clear areas of impairment noted in patients with MCI and going on to develop AD. In this case, no measures from the NIH Toolbox-Cognition came into play, most likely because One Card Learning explained the primary difference across the groups in terms of learning impairment. Then, the degree divergence is a measure that emphasizes the existence of high degree vertices. A loss of such vertices is commonly observed in neurological disorders [52]. Moreover, the importance of One Card Learning may be highlighting the associations not only found in terms of frontal connections but also highlighting the finding of lower degree divergence in MCI patients indicating more linear relationships in those patients. Last but not least, these three selected features came from three different domains, further suggesting that it is promising to combine measurements of functional connectivity, graph topology and cognition together for diagnosing MCI.

Our study has several strengths. First, we used the PLI as a measure of functional connectivity. The PLI has been shown to reduce the effects of volume conduction and active reference electrodes [39]. Second, to identify brain graph topology in MCI and control African American elders, we applied both the traditional measures, which are commonly used in the literature, and the MST approach that allows unbiased identification of graph changes. Also, our study indicates that the MST approach is sensitive enough to detect differences between healthy and MCI groups and supports previous research [25]. Third, statistical results in this study were assessed using permutation tests together with the false discovery rate for multiple comparisons. In this way, we achieved more reliable results.

The current report should be interpreted not only in the context of its strengths but also in the context of possible limitations. This convenience sample was recruited from ostensibly normal controls in the community (with memory self-concerns) and actually, the finding of the high percentage of MCI in the population was not expected. Additional research will be needed to replicate our findings on other ethnic representative samples of non-African Americans and may provide opportunities to examine additional factors relevant to differences in cognitive aging. In this regard to an additional limitation, within our sample of older African Americans, 90% were females. Unfortunately, this is a common issue in research [53].

In conclusion, our findings demonstrated that African American elders in a community setting find EEG and computerized testing acceptable and results are promising in terms of differentiating between healthy controls and persons with MCI. Utility in identifying persons at risk for MCI and the cognitive decline appears more sensitive when both electrophysiological and objective cognitive test measures are combined. This procedure may be especially important when considering a community sample, where individuals are more reluctant to take part in studies with large demands of time, as well as being potentially intrusive in terms of their evaluation approaches. Basic EEG and sensitive computer-based cognitive assessments may be critical in screening large numbers of persons living in the community to highlight those for whom more costly and complex amyloid and tau imaging approaches would be appropriate. Also, our approach holds much promise for its ability to identify patients who may be good candidates for interventional clinical trials for MCI and neurodegenerative disease while also screening out healthy controls without clear cognitive impairment.

## Supporting information

S1 Table Spearman's correlations in all 40 subjects. (DOCX)
