# Supplementary material for: Effective differentiation of mild cognitive impairment by functional brain graph analysis and computerized testing
Source: PLoS One. 2020 Mar 16;15(3):e0230099. doi: 10.1371/journal.pone.0230099 (PMC7075594; doi:10.1371/journal.pone.0230099)
Supplement: S1 Table — (DOCX) [file pone.0230099.s001.docx]

S1 Table: Spearman's correlations in all 40 subjects.

| Measure | characteristic path length | | clustering coefficient | | |
| --- | --- | --- | --- | --- | --- |
|  | R | P value |  | R | P value |
| $D^{*}$ | 0.0316 | 0.8467 |  | -0.4575 | **0.0030** |
| $l^{*}$ | 0.0567 | 0.7282 |  | 0.6751 | <**0.0001** |
| $D^{*}$, normalized diameter; $l^{*}$, normalized leaf fraction. | | | | | |
